# The impact of maturation level, not chronological age, on attentional control: implications for sports injury prevention in female adolescents

**DOI:** 10.1186/s13102-024-00984-5

**Published:** 2024-09-18

**Authors:** Adam Grinberg, Ivana Hanzlíková, Michal Lehnert, Reza Abdollahipour

**Affiliations:** 1 Department of Community Medicine and Rehabilitation, Umeå University, Umeå, Sweden; 2 Department of Physiotherapy, Faculty of Physical Culture, Palacký University Olomouc, Olomouc, Czech Republic; 3 Department of Sport, Faculty of Physical Culture, Palacký University Olomouc, Olomouc, Czech Republic; 4 Department of Natural Sciences in Kinanthropology, Faculty of Physical Culture, Palacký University Olomouc, Olomouc, Czech Republic

**Keywords:** Flankers test, Puberty, Selective attention, Response inhibition, ACL, Neurocognitive test, Reaction time, Injury prevention

## Abstract

**Background:**

Non-contact injuries are highly prevalent among young athletes and occur particularly in situations that require fast decision making and divided attention. Administering relevant neurocognitive tests could help identify deficiencies in these cognitive abilities and thus potentially mitigate injury risk. However, processes such as selective attention and response inhibition might depend to some extent on the athlete’s maturation stage. We aimed to examine the effect of maturation on selective visual attention and response inhibition among adolescent volleyball players.

**Methods:**

In this cross-sectional study, 52 female adolescents (age 12.3 ± 2.1 years) performed an Eriksen Flankers task. Participants were divided into subgroups based on their estimated adult stature, using the Khamis & Roche method: Pre-pubertal (PRE; *n* = 13, age: 9.9 ± 1.3), early-puberty (EPUB; *n* = 7, age: 10.5 ± 0.6), mid-puberty (MPUB; *n* = 8, age: 12.6 ± 0.8) and late puberty (LPUB; *n* = 24, age: 14.1 ± 0.9). Analyses of covariance (ANCOVA) were performed on congruent and incongruent reaction times (RT), with corresponding success rates (% correct responses) as covariate. Flanker interference effect was tested using ANOVA. Correlations were further examined between the dependent variables and participants’ chronological age.

**Results:**

There was a significant group effect, with PRE demonstrating longer RT compared with LPUB (*P* < 0.001) for both congruent and incongruent RT. Moderate negative correlations were observed between age and RT (R_p _= -0.695, R_p _= -0.614 for congruent and incongruent RT, respectively) and low positive correlations between age and incongruent success rate (R_s _= 0.318). Low to moderate correlations were also observed within the LPUB group for RT (R_p _= -0.431–-0.532) and success rate (negative R_s _= -574 for congruent and positive R_s _= 0.417 for incongruent). There were no group differences nor age associations with interference effect.

**Conclusions:**

Our findings indicate that information processing and selective visual attention are superior at late maturation compared with early maturation among female adolescents. The same cannot be said for response inhibition, which did not differ between maturation groups. Similar tendencies were observed with regards to chronological age, but not entirely explained by it. Maturation level, rather than chronological age, should guide practitioners during sport participation and injury prevention programs for young athletes, whose neurocognitive abilities are not yet fully developed, placing them at risk for non-contact injuries.

## Introduction

Major sporting injuries, e.g., anterior cruciate ligament (ACL) tears, are a main concern among athletes and practitioners, with non-contact mechanisms accounting for more than 50% of injuries during team ball sports [1] and a significantly higher risk for injury among females in general [2, 3] and female adolescents in particular [4]. In addition to risk-associated tasks such as landing and cutting manoeuvres [5], a staple of non-contact injuries is the presence of a distractor (e.g., another player in close proximity) [6–10] as well as a need for attentional control – the capacity to choose what to attend to and what to ignore – and fast decision making, both have been shown to influence lower limb biomechanics [11]. Excessively high cognitive demands, which are frequent in the dynamic environment of team ball sports, are thus intensified due to an abundance of visual and somatosensory cues in a rapidly changing environment. Consequently, during game situations, an athlete’s attentional pool may become overloaded, potentially exceeding their capacity and leading to injuries [12]. An athlete must carefully divide their attention in order to cope with game-related demands (e.g., other players, field of play), while maintaining adequate joint stability during complex movements [13]. Selective visual attention, the ability to filter out irrelevant visual information [14], is prudent for athletes who are faced with conflicting information and are required to rapidly react under pressure. Practitioners may need to get a clearer picture of an athlete’s capability to focus their attention on task-relevant information (while gating out the irrelevant) and consequently select appropriate responses. Testing attentional resources along with additional neurocognitive constructs could subsequently help identify potential deficits that may contribute to the susceptibility of an athlete to getting injured.

The rate of ACL injuries is particularly high among adolescents compared to adults [1]. When considering neurocognitive function, previous evidence clearly shows age-dependent functioning in various neuropsychological domains such as attention, executive function, memory and learning, with significant developments occurring at preadolescent [15] and adolescent [16] stages. It has additionally been suggested that maturation stage should be considered in this context, rather than chronological age of child athletes [17, 18]. Given that adolescents mature at different rates [19], it is crucial to investigate the impact of maturation/growth level on neurocognitive abilities, as it may provide valuable insights into the vulnerability of individuals at different developmental stages to non-contact injuries. This consideration is, however, seldom made, leaving practitioners with insufficient information to incorporate neurocognitive training into injury prevention programs for adolescent athletes.

The Eriksen Flanker task [20] is a reliable (test-retest ICC = 0.82–0.86 [21]) and easily administered choice reaction time test to assess an individual’s selective attention and their capability to suppress inappropriate responses to a given stimulus (i.e., response inhibition). The test involves repeated presentations of a central target letter surrounded by flanker letters that can be either congruent or incongruent with the central one. The participant is then required to react to the central target by pressing an appropriate predefined button, while ignoring the surrounding stimuli. Previous work has shown that response inhibition, as measured by Flanker test interference effect, is highly dependent on age, with full maturity suggested to not be reached until the age of 11 [22]. This finding, to some extent, was consistent with a prior study, which reported age-dependent improvements in flanker interference effect on error rates [23]. However, none of the previous studies considered pubertal maturation stage when grouping children in their respective cohorts, which given the potential variability in children’s neurocognitive development, warrants additional investigation. Moreover, relying solely on chronological age can potentially obscure important differences in cognitive abilities. This may negatively impact slowly developing adolescents who are expected to perform at the same as their age-matched peers. In the current study, we administered the Flanker task among a cohort of female volleyball players of different maturation levels, estimated based on predicted adult stature using the method developed by Khamis & Roche (1994) [24]. This approach utilizes a regression equation incorporating current stature, current body mass, and the average stature of both biological parents to predict the eventual adult stature of the child. We aimed to assess the effect of maturation status on reaction times (RT), success rate and flanker interference effect, with implications for future non-contact injuries among physically active female adolescents. We hypothesised that more mature adolescent would demonstrate better RT and success rates. As a secondary aim, we assessed these same outcomes in relation to chronological age. We hypothesised similar tendencies with respect to age, with better Flankers test performance among older participants.

## Methods

### Study design

This was a cross-sectional study, performed in a controlled testing environment setting within the sports hall of the BALUO Application Centre, Olomouc, Czech Republic. The study received approval from the Ethics Committee of the Faculty of Physical Culture, Palacký University Olomouc (reference number: 15/2023) and was conducted in accordance with the Declaration of Helsinki. Written informed consent documents were obtained from all guardians of the participants, after providing them with detailed information about the potential risks associated with the testing procedures.

### Participants

Recruitment of test participants took place during February 2023. The necessary sample size was predetermined using G*Power version 3.1.9.7 [25]. The analysis was based on effect size from previous study examining the differences of reaction time between early and late adolescent groups [26], a statistical significance level α = 0.05 and power 1-β = 0.95. The result indicated a required sample of 40 participants. To accommodate a potential 50% disagreement in study participation, as well as potential withdrawals and missing data, we approached 80 participants. All participants were female volleyball players 7 to 15 years old from the same club, engaged in training three times a week. Participation required meeting the following inclusion criteria: (a) active engagement in competitive volleyball; (b) absence of significant orthopaedic injuries (e.g., sprains, fractures, and tears) for a minimum of three months before the study commencement; (c) absence of any pain that might restrict participation. As our target population were adolescents with typical development, individuals with a diagnosed neurodevelopmental disorder were excluded. Participants were recruited using the following process: a researcher approached the main coach who then approached the other divisional coaches to explain the study. They were then responsible for primary screening of athletes. Eligible athletes were given an information sheet describing the aim of the research and the testing requirements. Out of a total of 80 participants contacted, 56 agreed to participate in the research. Three participants were then excluded due to missing data on biological parents’ height, which was crucial for group allocation (see below), and an additional participant was later excluded for being a statistical outlier, which resulted in a final sample size of 52 participants.

### Testing procedures

#### Anthropometrics and maturity measures

Data collection occurred during two consecutive testing days in the month of March 2023. It included an extensive test battery comprised comprising performance-related functional tests (e.g., drop-jumps, dynamic balance) and testing for injury-related fisk factors. Maturity calculations according to Khamis & Roche (1994) [24] were performed with the aid of Microsoft Excel spreadsheets created by Towlson (2020) [18] and were used to define the study groups. The assessment categorised participants into different maturation stages based on the percentage of predicted adult stature attained. Specifically, ranges of < 85%, ≥ 85% to < 90%, ≥ 90% to < 95%, and ≥ 95% were indicative of pre-pubertal (PRE; *n* = 13), early pubertal (EPUB; *n* = 7), mid-pubertal (MPUB; *n* = 8) and late-pubertal (LPUB; *n* = 24) maturation stages, respectively [17].

### Flankers test

The Flanker test was employed in this study to evaluate selective visual attention and response inhibition, given its established reliability and appropriateness for the target population of young females [27]. Testing was conducted in a laboratory setting, with only one participant and the researcher present to maintain an undisturbed environment. To ensure a comfortable and conducive testing process, several adjustments were made: The chair height was adjusted to align the table with the participants’ elbow level, and if necessary, a box was placed under their feet to provide stable and supported foot positioning. The computer screen was positioned at an arm’s length from the participants, and the first row on the screen was adjusted to align with the participants’ eye height.

Participants were instructed to sit comfortably and position their left index finger on the key “A” and their right index finger on the key “L,” awaiting the commencement of the test. Subsequently, standardized instructions were provided to each participant. The target stimulus was consisted of one of four letters. The letters X and C had to be responded by hitting the A button of the keyboard, while the letters V and B had to be responded to with the L button. The response was dictated by the central letter alone, while ignoring the surrounded letters, which could be either congruent or incongruent. A practice trial was performed prior to the actual test and continued until the participant completed ten correct trials (correctly pressed the appropriate key). Following the practice trial, headphones were placed on the participant to minimize any potential disruptions caused by external noise sources. To minimize any potential observer effects, the examiner positioned themselves out of the participant’s visual field.

### Statistical analysis

Four dependent variables were defined for the main analysis: Congruent / incongruent RT and success rate. The latter was calculated as the percentage of correct trials from all trials in the corresponding condition (i.e., inversely reflecting error rate). Consequently, flanker interference effect was calculated for each participant, for both RT and success rates. This was done by subtracting the mean congruent values from the mean incongruent values.

Data are presented using arithmetic mean and standard deviation. The Shapiro-Wilk tests and histograms were applied on each dependent variable to assess data distribution. Data was further tested for extreme outliers using boxplot with 3X interquartile range rejection criterion [28]. Incidentally, one participant (post-pubertal group) was excluded from the analysis for having significantly high RT values. For the main analysis (done separately for congruent and incongruent conditions), one-way analyses of covariance (ANCOVA) were employed, with four-level between group factor (PRE, EPUB, MPUB and LPUB) and corresponding success-rate as covariate. A similar analysis but without a covariate was performed for interference effect. We applied Bonferroni corrections for multiple comparisons. Effect-size estimates were assessed using η_p_^2^, interpreted as: small 0.10–0.29, medium 0.30–0.49 and large ≥ 0.50 [29]. Success rates were also analysed independently for group differences using Kruskal-Wallis H test.

In addition, Pearson / Spearman correlation tests were performed between participant age and the dependent variables. This was done both on the entire group as well as withing groups. Correlation sizes were interpreted as negligible (0-0.3), low (0.3–0.5), moderate (0.5–0.7), high (0.7–0.9) or very high (0.9-1) [30]. A sensitivity power analysis was conducted using G*Power version 3.1.9.7 [25]. The analysis was based on Pearson’s correlation coefficient, with a statistical significance level of α = 0.05, power of 1-β = 0.80, and a sample size of *n* = 56. The outcome revealed a minimal detectable value for the correlation coefficient of R_p_ = 0.370.

All statistical analyses were performed using the Statistical Package for the Social Sciences (version 28, IBM SPSS Statistics, Armonk, New York, USA) with significance level of α ≤ 0.05 set a-priory.

## Results

Participant demographics and background information are presented in Table 1.

**Table 1:** Participants background information presented as mean (standard deviation)

	PRE (*n* = 13)	EPUB (*n* = 7)	MPUB (*n* = 8)	LPUB (*n* = 24)	*P*-value (main effect)
Age (yrs)	9.9 (1.3)	10.5 (0.6)	12.6 (0.8)	14.1 (0.9)	< 0.001
Body height (cm)	143.7 (11.0)	147.3 (3.2)	161.5 (6.0)	166.8 (7.1)	< 0.001
Body mass (kg)	36.8 (12.4)	42.4 (8.3)	47.0 (6.9)	59.1 (8.9)	< 0.001
Predicted height (cm)	179.1 (12.4)	169.5 (2.3)	172.4 (6.1)	170.1 (5.9)	0.016
APHV (age)^1^	11.6 (0.3)	11.6 (0.5)	11.8 (0.6)	12.1 (0.6)	0.014
Khamis-Roche (%)^2^	80.3 (4.6)	86.9 (1.4)	93.7 (1.3)	97.7 (1.5)	< 0.001

^1^ Anticipated peak age velocity – the age when fastest growth is expected. Calculated based on height measurement and the corresponding age

^2^ Percentage of the current height from the predicted height

### Maturation effect

**Fig. 1:**
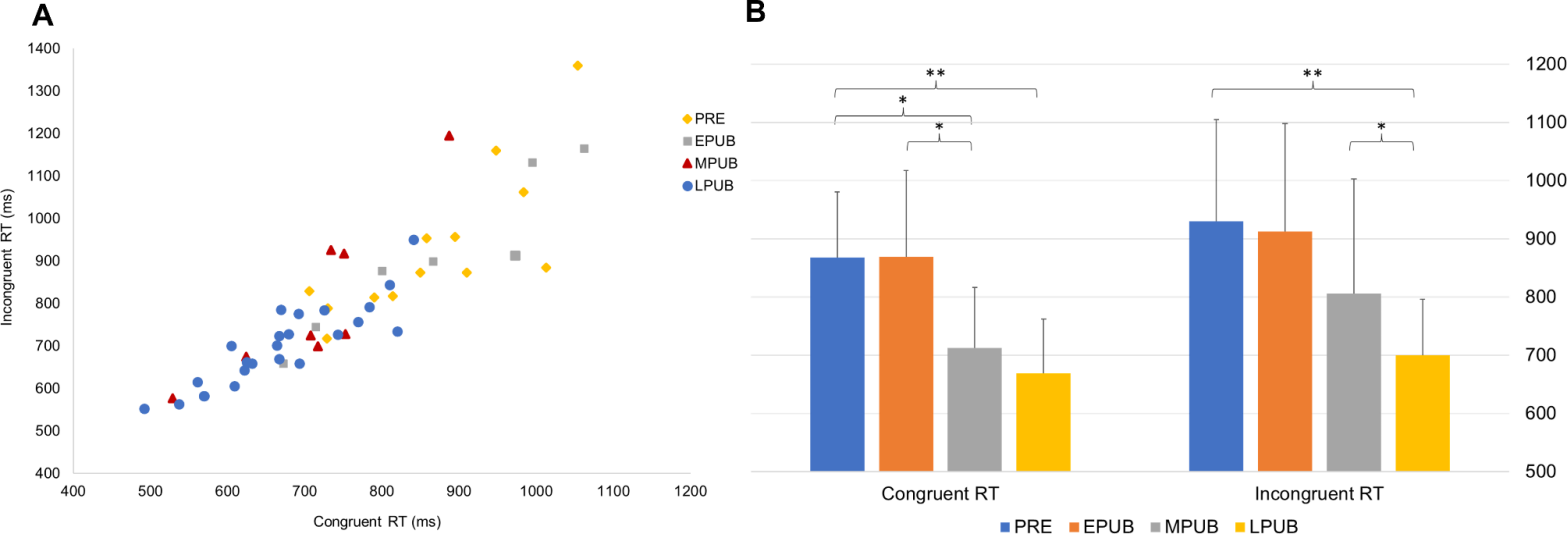
Reaction time comparison across maturation.(**A**) Scatter plot of congruent and incongruent reaction time (RT) for each maturation group.(**B**) Mean RT values. A clear improvement can be observed with maturation, both in congruent and incongruent RT values. *Abbreviations*: PRE, pre-pubertal; EPUB, early pubertal; MPUB, mid-pubertal; LPUB, late pubertal

Significant maturation effect was observed for both congruent (F [4, 47] = 13.479, *P* ≤ 0.001, η_p_^2^ = 0.534) and incongruent (F [4, 47] = 7.543, *P* ≤ 0.001 η_p_^2^ = 0.391) reaction times (Fig. 1). Pairwise comparisons revealed significantly lower RTs (both congruent and incongruent) for the LPUB compared to PRE and EPUB groups (∆_mean_ = 199-230ms, *P* < 0.002; ∆_mean_ = 200-213ms *P* < 0.03, for PRE and EPUB respectively). Significantly shorter congruent RTs were also observed for MPUB compared to PRE (∆_mean_ = 155ms, *P* = 0.010) and to EPUB (∆_mean_ = 156ms, *P* = 0.017). While success rate, as covariate, significantly influenced the congruent RT comparison (*p* = 0.005, η_p_^2^ = 0.534), as an independent variable, there were no group effects. There were also no significant group differences in RT or success rate interference effects.

### Age effect

**Fig. 2:**
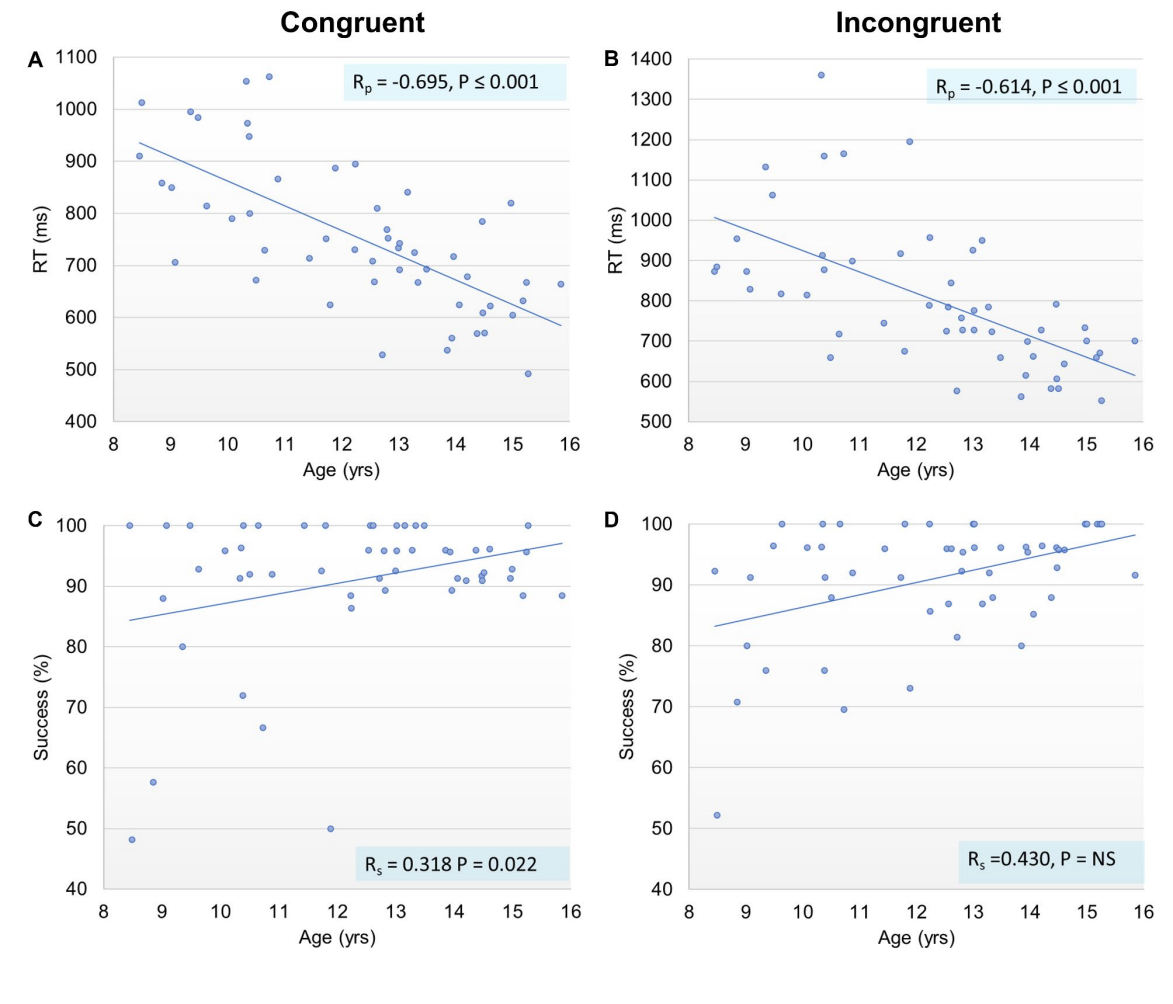
Correlation analysis between age with Flankers test outcome variables. (**A**-**B**) Moderate negative correlations were observed for reaction times (RT); (**C**-**D**) Note: low positive correlation coefficient seen for congruent success rate, did not pass the sensitivity threshold

Moderate negative correlations were observed between age and reaction times (R_p_ = -0.695, *P* ≤ 0.001 for congruent and R_p_ = -0.614, *P* ≤ 0.001 for incongruent RT; Fig. 2). Age was not associated with flanker interference effects (both for RT and success rate) and had low positive correlation with incongruent success rates (R_s_ = 0.318, *P* = 0.022). In a further analysis, age correlations within each subgroup were tested. Only in the LPUB group, significant low to moderate negative correlations were observed between age and RT (R_s_ = -0.431, *P* = 0.035 for congruent and R_s_ = -0.532, *P* = 0.007 for incongruent RT) and between age and success rate (R_s_ = -0.574, *P* = 0.003 for congruent and R_s_ = 0.417, *P* = 0.043 for incongruent success rate).

## Discussion

To our knowledge, this was the first study to evaluate the effect of maturation status, rather than chronological age, on attentional control and response inhibition. Our results demonstrate a clear positive effect of maturation on choice reaction times, both congruent and incongruent, which were shorter in late maturation compared to early and pre-maturation. A similar relationship was observed for chronological age, with negative moderate correlations between age and reaction times. Congruent success rate was positively correlated with age, although not significantly different between maturation groups. Neither maturation level nor age were shown to affect flankers’ interference.

Shorter reaction times observed in LPUB participants may suggest a pivotal role of pubertal development in influencing cognitive performance, which tends to culminate towards full puberty. This finding is supported by Luna et al. [16], who demonstrated that processing speed and voluntary response suppression reach adulthood level at around ages of 14–15. In the current study, the mean age for the LPUB group was 14.1, although lower aged participants (12.6) were also classified as late pubertal stage according to maturity status and incidentally had lower RTs compared to the older participants in the group. In fact, correlations with age within the LPUB group were lower compared to the entire cohort age correlations. There was also an absence of correlations between age and reaction times in less mature groups, while LPUB success rate correlations were mixed, with negative correlations for congruent rates and positive correlations for incongruent. Together, these findings suggest that age alone may not be used to fully explain the measured constructs, though this practice is common [16, 31, 32]. In the current study, we used the Khamis-Roche method which has been found to provide a superior precision in maturity estimation compared to other methods which utilise various equations [18] Practically, such estimation is simple to apply by practitioners among adolescents, as it does not rely on direct measures such as skeletal age [33], dental maturity [34] or secondary sex characteristics [35]. Our findings carry important implications for practitioners working with adolescent athletes. Matching team assignments and tailoring training programs is currently based on chronological age. We propose, however, that maturation status should be taken into consideration when cognitive abilities are a requirement for a particular sport. Late-maturing individuals, despite their age, may face challenges in tasks whereas choice reaction is a major component (e.g., selecting where to pass a ball during a game), suggesting a potential disadvantage that warrants specific attention in training sessions [36].

While a positive correlation of incongruent success rate with age contrasted with the absence of significant differences for this outcome measure in relation to maturation subgroups, the observed correlation of 0.318 was below the minimal detectable value based on our sensitivity analysis. This strengthens the notion that success rate as an outcome might not be related to maturation stage nor to age. Consequently, it may suggest that participants prioritised accuracy over speed, resulting in similar accuracy rates between maturation subgroups but varying reaction times. It can also mean that this particular outcome is not sensitive enough with a clear ceiling effect that is achieved in relatively early development. It has previously been suggested that these two outcomes might benefit from a joint analysis rather than processed independently from one another [37]. Indeed, success rate accounted for some of the variability for congruent RT group difference, though not incongruent.

In the current study, neither maturation nor age were seen to be related to the flanker interference. It is likely that focusing on a central letter which is morphologically distinct than the alternative counts as a low perceptual task. Therefore, a high interference effect that was observed regardless of age might suggest that participants were potentially equally prone to distractibility [38]. While visual information processing time seemed to be different between groups, the interference effect is an individually normalised differential between congruent and incongruent RTs. Therefore, distractibility and, by extension, the interference effect could depend primarily on the perceptual load which did not exceed the capacity of even the pre-maturation participants. It is noteworthy that the Flankers test used in the current study included an element of working memory and thus, cognitive control. In this sense, a high cognitive, rather than perceptual, load was a staple of test performance. According to the load theory of attention [38], tasks with low perceptual load would typically result in greater distractibility compared to tasks that involve a high perceptual load. This is because when processing a task-relevant stimulus that is considerably high and closer to the individual’s perceptual capacity limits, processing distractors would theoretically result in exceeding this capacity [38].

Incidentally, caution is warranted when interpreting the findings of this study regarding the influence of puberty on inhibitory control. Despite the absence of any discernible effect of puberty on resistance to distractor interference in the Flanker test, puberty did exhibit an influence on selective attention, evident in reaction times when compatible and incompatible flankers were presented. It is essential to distinguish between resistance to distractor interference and inhibition. The former refers to the ability to resist or resolve interference from irrelevant external information, while the latter implies a specific mechanism or explanation for that effect [39–41]. Thus, the complexity of the interference effects suggests that other cognitive processes may be involved beyond straightforward inhibitory mechanisms [41]. Moreover, assessments such as the Flanker test, stop-signal task [42] or Stroop task [43] do not exclusively measure inhibition, as different task demands for each test may influence the shared features or patterns that can be linked to inhibitory processes [40, 44]. Additionally, it is worth noting that the inhibitory requirements for these repeated tasks may undergo changes upon task repetition, as highlighted in previous studies [41, 45]. Therefore, future research should carefully consider the specific features of inhibitory tests when attempting to assess the association between puberty and inhibitory control.

On another level, the question remains whether Flanker test performance is reflective of real-life sport situations that involve high perceptual load due to abundant peripheral stimuli. In these open skill situations, athletes interact with opponents as well as collaborate with their peers [46]. While evidence exists that sporting performance may be influenced by an athlete’s ability to supress their responses [46], the relationship between inhibitory control and sports performance is complex. Furthermore, rather than just ignoring irrelevant stimuli, spatial covert attention as a construct involves attending to peripheral information during complex tasks, not merely blocking it. We propose that even simple choice RT tasks with a response suppression element are to some extent a piece of the puzzle when investigating attentional control during real-life scenarios. Once more, this is particularly relevant for open-skill athletes, who have shown to demonstrate superior inhibitory control compared to closed-skill athletes [47]. In designing training routines for young athletes, it is crucial for coaches and sport practitioners to take into account the maturation level of the athletes. Specifically, practitioners should recognize that regardless of chronological age, less matured athletes may have different attentional capacities, adjust training protocols accordingly and align them with the athletes’ developmental neurocognitive capabilities. Although our cohort consisted of non-injured individuals, our findings do have potential implications for injury prevention, given that a majority of non-contact ACL injuries occur in situations that require fast decision making [9, 11]. Previous work has shown decreased neurocognitive performance among athletes that had suffered non-contact ACL injury compared to non-injured controls [48]. Although the risk of non-contact injuries is indeed significant during team sports [1], the latter evidence does not necessarily indicate a causal relationship, since various deficits might relate to post-injury neuroplasticity [49]. While our cohort was not consisted of injured persons, they have been active in team sports. In a recent work, Gonçalve et al. [50] demonstrated a positive association between maturation level (determined using the Khamis-Roche method) and peripheral perception among young soccer players, which was suggested to translate to superior efficiency in perceiving the complex environment of real-life game situations and subsequently, to better tactical performance.

Our study has several limitations, one of which is the uneven group sizes. The power analysis conducted prior to the study was based on the assumption of evenly distributed groups. The actual distribution thus led to a reduction of statistical power in the less matured groups. We argue, however, that smaller samples might suffice for early maturation groups given less variability in neurocognitive function within this demographic. Moreover, the most significant differences in RT were observed at late maturation, aligning with existing literature [16]. Furthermore, we initiated our study with a prior calculation using a high beta (1-β = 0.95) and recruited more participants than initially required. A post hoc power analysis using G*Power version 3.1.9.7 [23] and our results revealed that the achieved power was 1-β = 0.89 for the maturation effect on congruent RT and 1-β = 0.61 for maturation effect on incongruent RT, which seemed sufficient. Still, future studies are encouraged to confirm our findings by including larger cohorts in earlier maturation stages as well. Secondly, due to inherent variability between individuals, neither age nor maturation can be attributed as the only determinants of long/short reaction times. In that sense, longitudinal study designs are warranted to further establish the effect of maturation on neurocognitive function. Thirdly, we did not account for the potential impact of years of open-skill training on executive function in our older participants. While this trend has been systematically observed in typical children, particularly in the domain of working memory [51], no such influence was reported regarding inhibitory control. This suggests a minimal effect on the current findings. Finally, our cohort consisted of non-injured adolescents. Consequently, any inferences regarding the risk of non-contact injuries remain speculative. Future work should incorporate longitudinal follow-up documentation of potential injuries, to enhance our understanding of the risk and its relation to neurocognitive performance.

## Conclusion

Our results demonstrate that maturation stage has a clear effect on visual information processing and selective attention among female adolescents active in team sports. In contrast, response inhibition did not seem to be affected by maturation level. These tendencies were comparable to an age effect, although age alone did not correlate with test performance within all maturation groups. Sport practitioners are encouraged to consider maturation level when developing training and injury prevention protocols among young athletes. Such practice could facilitate an individualised approach that is more compatible with their developmental neurocognitive capabilities.

## Data Availability

The datasets used and/or analysed during the current study are available from the corresponding author on reasonable request.

## Abbreviations

ACL: anterior cruciate ligament
ANCOVA: analysis of covariance
ANOVA: analysis of variance
EPUB: early pubertal group
LPUB: late pubertal group
MPUB: mid-pubertal group
PRE: pre-pubertal group
RT: reaction time
